# Effectiveness of High-Intensity Interval Training and Continuous Moderate-Intensity Training on Blood Pressure in Physically Inactive Pre-Hypertensive Young Adults

**DOI:** 10.3390/jcdd9080246

**Published:** 2022-08-03

**Authors:** Anil T John, Moniruddin Chowdhury, Md. Rabiul Islam, Imtiyaz Ali Mir, Md Zobaer Hasan, Chao Yi Chong, Syeda Humayra, Yukihito Higashi

**Affiliations:** 1 Faculty of Health Sciences, Lincoln University College, Petaling Jaya 47301, Selangor, Malaysia; anil1452@gmail.com; 2 College of Physiotherapy, Dayananda Sagar University, Bengaluru 560111, India; 3 Faculty of Medicine, AIMST University, Bedong 08100, Kedah, Malaysia; moniruc@yahoo.com; 4 Department of Public Health, Daffodil International University, Dhaka 1341, Bangladesh; 5 Department of Public Health, Independent University Bangladesh, Dhaka 1229, Bangladesh; rabiulislamjuphi@gmail.com; 6 Department of Physiotherapy, Faculty of Medicine & Health Sciences, Universiti Tunku Abdul Rahman, Kajang 43000, Selangor, Malaysia; 7 School of Science, Monash University Malaysia, Bandar Sunway 47500, Selangor, Malaysia; mdzobaer.hasan@monash.edu; 8 General Educational Development, Daffodil International University, Dhaka 1341, Bangladesh; 9 Faculty of Medicine & Health Sciences, Universiti Tunku Abdul Rahman, Kajang 43000, Selangor, Malaysia; chaoyichong41@gmail.com; 10 Department of Public Health, Faculty of Allied Health Sciences, Daffodil International University, Dhaka 1341, Bangladesh; syedahumayra@gmail.com; 11 Department of Regeneration & Medicine, Research Center for Radiation Genome Medicine, Research Institute for Radiation Biology & Medicine, Hiroshima University, Hiroshima 739-8511, Japan; yhigashi@hiroshima-u.ac.jp; 12 Division of Regeneration & Medicine, Hiroshima University Hospital, Hiroshima 739-8511, Japan

**Keywords:** high-intensity interval training, continuous aerobic training, systolic blood pressure, diastolic blood pressure, pre-hypertension

## Abstract

The likelihood of pre-hypertensive young adults developing hypertension has been steadily increasing in recent years. Despite the fact that aerobic exercise training (AET) has demonstrated positive results in lowering high blood pressure, the efficacy of different types of AET among pre-hypertensive young adults has not been well-established. The objective of this study was to evaluate the effectiveness of high-intensity interval training (HIIT) and continuous moderate-intensity training (CMT) on the blood pressure (BP) of physically inactive pre-hypertensive young adults. In total, 32 adults (age 20.0 ± 1.1 years and BMI 21.5 ± 1.8) were randomly assigned to three groups: HIIT, CMT and control (CON). The HIIT and CMT groups participated in 5 weeks of AET, while the CON group followed a DASH diet plan only. The HIIT protocol consisted of a 1:4 min work to rest ratio of participants, at an 80–85% heart rate reserve (HR-reserve) and a 40–60% HR-reserve, respectively, for 20 min; the CMT group exercised at 40–60% of their HR-reserve continuously for 20 min. In both the HIIT and CMT groups, systolic blood pressure (SBP) (3.8 ± 2.8 mmHg, *p* = 0.002 vs. 1.6 ± 1.5 mmHg, *p* = 0.011) was significantly reduced, while significant reductions in the diastolic blood pressure (DBP) (2.9 ± 2.2 mmHg, *p* = 0.002) and mean arterial pressure (MAP) (3.1 ± 1.6 mmHg, *p* < 0.0005) were noted only in the HIIT group. No significant differences in SBP (−0.4 ± 3.7 mmHg, *p* = 0.718), DBP (0.4 ± 3.4 mmHg, *p* = 0.714), or MAP (0.1 ± 2.5 mmHg, *p* = 0.892) were observed in the CON group. Both HIIT and CMT decreased BP in physically inactive pre-hypertensive young adults; however, HIIT yielded more beneficial results in terms of reducing the SPB, DBP and MAP.

## 1. Introduction

Hypertension is considered to be one of the main precursors of cardiovascular diseases (CVDs) and has been linked to 7.7 million deaths globally [1,2]. The Seventh report of the Joint National Committee on Prevention, Detection, Evaluation, and Treatment of High Blood Pressure (JNC7) defined pre-hypertension as a systolic blood pressure (SBP) of 120 mmHg to 139 mmHg and a diastolic blood pressure (DBP) of 80 mmHg to 89 mmHg [3]. Pre-hypertensive people are at an increased risk of acquiring hypertension, and it has been estimated that those with blood pressure (BP) readings between 130 and 139/80 and 89 mmHg are twice as likely to develop hypertension than those with lower readings [4]. Modifiable risk factors of high BP can be controlled by active engagement in physical exercises [3,5]. To prevent the progressive rise in BP and cardiovascular diseases, the control of pre-hypertension and lifestyle modifications require special attention [6]. 

Studies have suggested that physical exercise is associated with substantial improvements in insulin sensitivity, augmented autonomic nervous system function and decreased vasoconstriction, which may prevent a pathological rise in BP [7,8]. Physical activity (PA) improves the release of growth factors from skeletal muscles into the bloodstream, stimulates angiogenesis, facilitates neurogenesis and induces endothelial cell proliferation with endothelial cell membrane permeability, thus leading to a substantial reduction in BP and the attenuation of hypertension symptoms [9,10,11].

Worldwide, 9% of premature mortality contributing to approximately 5.3 million deaths in 2008 occurred due to physical inactivity [12]. Regular PA is a well-established intervention for the prevention and treatment of several chronic diseases [13], and it has shown a significant effect on BP reduction [14]. Physical exercise has also been shown to improve various factors involved in the pathophysiology of hypertension [15,16,17,18], which can extenuate BP in both hypertensive and non-hypertensive adults [15,19]. Continuous moderate-intensity training (CMT) for at least 30 min or more is traditionally recommended for the prevention and treatment of high BP [13,20]. 

High-intensity interval training (HIIT) has been documented as a safe and effective training method for cardiac rehabilitation [21]. HIIT can be defined as a short burst of maximal effort interspersed by a few minutes of rest or active recovery, and it has been reported to be more effective than CMT for improving cardiorespiratory fitness in different populations [16,17,18,22,23,24]. HIIT, which consists of several bouts of high-intensity exercise (85–95% of HRmax) lasting 1 to 4 min interspersed with intervals of rest or active recovery [15,17,18], has been found to improve endothelial function and its markers [16,18], insulin sensitivity [18], markers of sympathetic activity [16,17], arterial stiffness [15,16], blood glucose and lipoproteins [18]. Despite these favorable outcomes, the efficacy of HIIT in reducing BP among pre-hypertensive young adults is not well established [25]. In addition, there is a scarcity of current literature comparing HIIT and CMT on BP in this particular population. Therefore, the primary purpose of this study was to determine the effects of HIIT and CMT on the BP of physically inactive pre-hypertensive young adults, and then to explore which type of exercise training is more efficient in lowering the BP of this population. To the best of the authors’ knowledge, this is the very first study to target pre-hypertensive young adults in Malaysia.

## 2. Materials and Methods

### 2.1. Study Setting and Subjects

This 5-week randomized–controlled trial was conducted in the Physiotherapy Centre at the Faculty of Medicine and Health Sciences in University Tunku Abdul Rahman, Sungai Long Kajang, Malaysia. G*power (F test) was used to calculate the sample size, based on the power analysis; a total of 42 participants were required for this study. The study subjects were reached through university portal, emails and posters for voluntary participation. Participants were recruited by convenience sampling as the study population required young adults with pre-hypertension. A total of 87 subjects were initially screened, out of which only 32 adults fit the eligibility criteria after they were administered the International Physical Activity Questionnaires (IPAQ), Physical Activity Readiness Questionnaire (PAR-Q+) and measurement of body mass index (BMI). Using the computer-generated numbers, study participants (22 males and 10 females) were randomly allocated to 3 groups: the HIIT group, CMT group and the control (CON) group.

Inclusion criteria comprised both genders (unmarried), aged between 18 and 25 years old, who were physically inactive with an SBP between 120 and 139 mmHg and/or DBP between 80 and 89 mmHg. Participants with a known history of respiratory illnesses, cardiovascular diseases, diabetes mellitus, overweight/obesity, psychological disorders, or musculoskeletal problems; those taking anti-hypertensive medications; and active smokers were excluded from this study. 

The protocol was based on the Helsinki Declaration Accord (World Medical Association for Human Subjects). Moreover, prior ethical clearance was obtained from the Universiti Tunku Abdul Rahman’s Scientific and Ethical Review Committee (U/SERC/77/20). Written informed consent was obtained from each participant after debriefing them about the benefits of the study, potential risks of muscle soreness, strict maintenance of data confidentiality and right to withdraw at any point from the study. 

### 2.2. Body Mass Index and Blood Pressure Measurement

In addition to assessing BMI during the screening process, it was also measured at baseline to ensure no abrupt changes in body weight before study initiation and that participants were within the normal BMI range (18.5–24.9). BMI was recorded by measuring the participants’ body weight in kilograms and dividing it by their height squared (kg/m^2^). The procedure was carried out early in the morning (8:30 am–9:00 am) using a calibrated seca 284 EMR (Hamburg, Germany) wireless measuring station for weight and height. Before measuring the BMI, participants were instructed to remove any excess clothing, and to stand upright and barefooted on the measuring machine. An average of 3 measurements for both weight and height were calculated to assess the BMI score.

Following the standard procedure, participants’ BP from the right brachial artery was measured using an automated digital BP monitor (OMRON SEM-1, Kyoto, Kansai Japan) in the morning between 9:15 am and 10:15 am after 5 min of rest in a chair [26,27]. Each participant’s right arm was supported on the table at heart level, and both SPB and DBP were measured 3 times with a 5 min interval between each measurement in order to obtain the most accurate result. If the differences between any of the 3 SBP and/or DBP readings were higher than 5 mmHg, the measurement was taken again after a 5 min interval, and the average reading with the least differences was taken into consideration. BP was measured at baseline before beginning the intervention and at the end of the 5-week intervention. The post-test measurement of BP was carried out in a similar way as recorded at baseline. In addition, the mean arterial pressure (MAP) was also estimated at baseline and at the end of intervention with the following formula:

MAP = DBP + 1/3(SBP − DBP)

### 2.3. Exercise Intervention Protocol

Before the first exercise session, the subjects’ heart rate (HR) was measured using a calibrated pulse oximeter (GIMA: Oxy-5-Plus Oximeter, Italy). The exercise HR (HRmax) of the participants in the HIIT and CMT groups was calculated using the newest age-based formula, [HRmax = 211 − (0.64 ∗ age)]. The exercise HR was then calculated using the Karvonen formula [Exercise HR = % of target intensity (HRreserve) + HRrest]. To prevent delayed-onset muscle soreness (DOMS) and to acclimatize all the physically inactive participants in both exercise groups to the exercise regimen, a 1-week familiarization period was provided with a total of 3 exercise sessions on alternate days. Participants in both the experimental groups performed a 5 min warm-up session followed by 20 min of continuous running on a treadmill (BH LK-G6700 Pro Action, St. Charles, MI, USA) without inclination at 40–60% of their HR-reserve. Before ending the exercise session, a 5 min cool-down was performed by all participants by walking on the same treadmill at their own comfortable pace. A pulse oximeter (GIMA: Oxy-5-Plus Oximeter, Gessate, Milan, Italy) was placed on the participants’ index finger during the PA to monitor their exercise HR, in addition to using the treadmill’s inbuilt heart rate monitor. Differences in the exercise HR in both the monitoring methods were negligible throughout the training protocol. After the familiarization period of 1 week, the HIIT group underwent 4 weeks of HIIT (3 times per week on alternate days excluding the weekends) consisting of 20 min of treadmill (BH LK-G6700 Pro Action, St. Charles, MI, USA) running with a 1:4 min work to rest ratio, an upper HR target at 80–85% of HR-reserve, and a lower HR target at 40–60% of HR-reserve. The CMT group continued with 4 weeks of the same exercise protocol on the treadmill, which was carried out in the familiarization period at an intensity of 40–60% of their HR-reserve. Exercise HR during these 4 weeks of aerobic exercise training (AET) for both groups were monitored in the same way as stated above in the familiarization program. The indication for the termination of the exercise sessions was in accordance with ACSM’s guidelines. It was not feasible to blind the participants or therapists, as they both knew the type of intervention being received and delivered, respectively, but outcome assessors were blinded to control the detection bias. 

The CON group did not participate in any exercise program; they were instructed to follow a Dietary Approaches to Stop Hypertension (DASH) diet and restrict their sodium intake (<100 mmol/day) according to the JNV VIII guidelines. In addition to the distribution of the guidelines given, participants in the CON group were reminded via telephone calls once weekly about the DASH diet and sodium restrictions to ensure that they were strictly following the guidelines.

**Figure 1:**
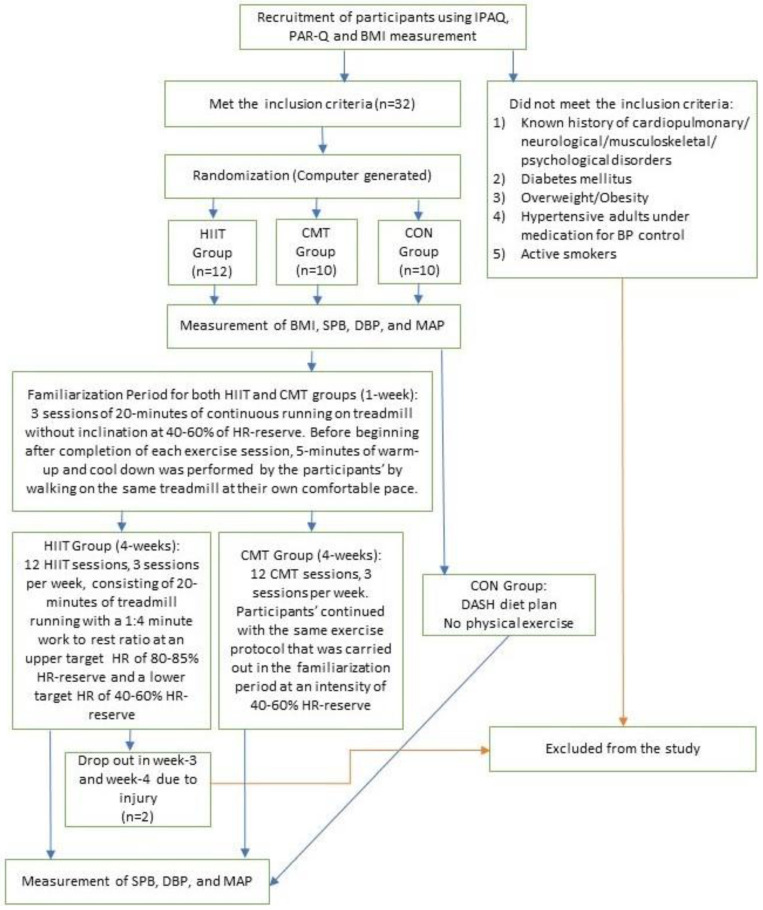
Flowchart of Trial: IPAQ = International Physical Activity Questionnaire; PAR-Q = Physical Activity Readiness Questionnaire; BMI = Body Mass Index; SBP = Systolic Blood Pressure; DBP = Diastolic Blood Pressure; MAP = Mean Arterial Pressure; HIIT = High Intensity Interval Training; CMT = Continuous Moderate-intensity Training; CON = Control; HR-reserve = Heart Rate Reserve.

All the participants in the 3 groups were instructed not to engage in any other form of PA during these 5 weeks to prevent any extraneous effect on the outcomes. In addition, to avoid the acute post-exercise effects on BP, participants were also instructed not to perform any exercise 24 h prior to the post-test BP measurement. In accordance with the CONSORT statement, a detailed description of this clinical trial is shown in Figure 1 below.

### 2.4. Statistical Analysis

The data were processed using the Statistical Package for Social Science (SPSS) version 26.0. The Shapiro–Wilk test was first performed to check the normality assumption of data as it is required to fulfil the conditions of a paired sample *t*-test. The Shapiro–Wilk test (Table 1) demonstrated that data were normally distributed (*p* > 0.05) in all 3 groups with respect to the SBP, DBP and MAP at baseline; therefore, these outcome measures were compared using the paired sample *t*-test to determine within-group differences. To evaluate between-group differences, a one-way ANOVA test was carried out. The conditions to conduct the one-way ANOVA test were fulfilled, in which each group represented the qualitative variables, and the dependent variables, SBP, DBP and MAP, were quantitative variables. A post hoc test was further employed to assess which group differed significantly from the other two groups after performing the one-way ANOVA test. Results are presented as the mean ± SD for all the outcome measures. All reported probability values were 2-sided, and a *p*-value of <0.05 was considered statistically significant. 

## 3. Results

### 3.1. Descriptive Statistics

At the beginning of this study, 32 participants were randomly assigned to the HIIT (6 males and 6 females), CMT (6 males and 4 females) and CON groups (10 males). Two participants dropped out of the HIIT group (both males) during the third and fourth weeks of training due to musculoskeletal injury. The mean ages of the participants in the HIIT, CMT and CON groups were 21 ± 0.8, 19 ± 1.3 and 21 ± 1.0, respectively. Similarly, the mean BMIs measured at baseline were 20.8 ± 1.9, 21.7 ± 1.6 and 22.0 ± 1.9 for the HIIT, CMT and CON groups, respectively. The BMI of the CON group was slightly higher than that of the other two groups, which was most probably due to the fact that all the participants in the CON group were males. 

### 3.2. Comparison within the Groups

Table 2 depicts that the CON group had the highest baseline and post-intervention SBP mean values of 127.93 ± 5.09 mmHg and 128.37 ± 5.32 mmHg, respectively. The HIIT group had the highest baseline DBP (78.57 ± 5.36 mmHg), and CMT group had greater post-test DBP (75.73 ± 4.26). At baseline, the MAP was highest in the CMT group (93.20 ± 2.89) and greater in the CON group (91.86 ± 4.18) post-test. 

Table 3 illustrates the results of the paired sample *t*-test. In the CON group, a mean difference of −0.43 (*p*-value = 0.718 > 0.05) for the SPB was observed, indicating a non-significant difference between the pre-SBP and post-SBP. For the DBP, a mean difference of 0.40 (*p*-value = 0.714 > 0.05) was found, showing no significant difference between the pre-DBP and post-DBP for CON group. Similarly, the MAP did not exhibit any significant difference (mean = 0.11, *p*-value = 0.892). For the CMT group, a mean difference of 1.57 (*p*-value = 0.011 < 0.05) was observed in terms of SBP, which was statistically significant. However, for the DBP, the mean difference between the pre- and post-tests was 1.50 (*p*-value = 0.161 > 0.05), depicting a non-significant difference between the pre-DBP and post-DBP in the CMT group. The MAP in the CMT group showed an insignificant reduction in the mean (1.49, *p*-value = 0.054). A mean difference of 3.76 (*p*-value = 0.002 < 0.05) was found in the HIIT group for the SBP and 2.93 (*p*-value = 0.002 < 0.05) for the DBP, indicating a statistically significant difference between the pre- and post-tests of both the SPB and DPB, respectively, in the HIIT group. A similar result was noticed found in MAP, with a significant mean difference of 3.05 (*p*-value < 0.0005).

### 3.3. Comparison between the Groups

For the SBP, the F-test (one-way ANOVA) result was 5.02 (*p*-value = 0.014 < 0.05) (Table 4). Therefore, it can be concluded that there were significant differences in the mean SBP across the three groups. However, for the DBP, the F-test statistic was 1.87 (*p*-value = 0.173 > 0.05), indicating a non-significant difference among the three groups. The MAP F-test was 4.76 (*p*-value = 0.017 < 0.05), showing a significant difference between the 3 groups.

Since the one-way ANOVA test showed significant differences in SBP and MAP across the three groups, a post hoc test (Tukey test) was performed to investigate which pairs of the groups were different in terms of the mean SBP and mean MAP. We found that the SBP mean difference in the HIIT and CMT groups was statistically insignificant (*p*-value = 0.282 > 0.05) (Table 5). However, we noticed a significant SBP mean difference between the HIIT group and the CON group (*p*-value = 0.010 < 0.05), but the SBP mean difference between the CMT and CON groups was statistically insignificant (*p*-value = 0.258 > 0.05). The MAP did not show any significant mean difference between the HIIT and CMT groups (*p*-value = 0.244 > 0.05) and between the CMT and CON groups (*p*-value = 0.337 > 0.05). However, a significant mean difference in MAP was seen between the HIIT and CON groups (*p*-value = 0.013 < 0.05). Hence, it can be deduced that HIIT is more effective in reducing the SBP, DBP and MAP compared to the CMT. 

## 4. Discussion

Earlier studies [28,29] broadly supported the improved cardiopulmonary benefits of HIIT over CMT. Nevertheless, no previous study has conspicuously explored HIIT and CMT outcomes in the pre-hypertensive young population by incorporating a comparator CON group with the DASH protocol. Thus, the research provided valuable insights into the field of physical therapy and significantly contributed to the current body of scientific literature. This study showed the beneficial effects of HIIT and CMT on the resting BP of physically inactive young adults with pre-hypertension. It is evident from the findings of the current study that both HIIT and CMT can reduce SBP significantly among pre-hypertensive young adults. The positive role of exercise training on BP can be perceived through its action on sympathetic activity, enhanced endothelial function and decreased oxidative stress, which cumulatively contributes to the prevention and treatment of hypertension [30]. In addition, PA may be accountable for reducing exercise-induced oxidative stress by producing an increased level of antioxidants, attenuating vascular and cardiac sympathetic activity, reducing serum vasoconstrictor factor levels and increasing endothelial dilating factors, which that consequently helps in lowering the peripheral vascular resistance and subsequently leads to improved BP [16,31]. A previous meta-analysis revealed that the two most prominent intervention protocols HIIT and CMT were effective in reducing SBP in adults with pre- to established hypertension [25]. Our findings correlate with a study that compared the effects of continuous and interval training in the management of hypertension, wherein researchers found a SBP reduction in both experimental groups (−16.4 ± 13.2 mmHg and −13.9 ± 12.6 mmHg, respectively) [32]. Similar results were also derived from the systematic review by Punia S et al. [33]. Our study revealed significant reductions in SBP after conducting 5 weeks of an AET (HIIT and CMT) program. Therefore, in addition to lowering SBP among the hypertensive population, HIIT and CMT can be useful tools in reducing the SBP among pre-hypertensive young adults.

The current study demonstrated a significant reduction in DBP among the participants undergoing the HIIT exercise protocol, whereas a non-significant reduction in DBP was observed among the CMT and control groups. In [34], it is suggested that HIIT demonstrated greater improvements in the endothelial function and arterial stiffness compared to CMT. This explains the increased BP reduction in the HIIT group as endothelium plays a pivotal role in the homeostasis and maintenance of vascular tonus, which may be a contributing factor in BP reduction. A recent randomized clinical trial also revealed similar results, where the authors found a significant reduction in SBP but a non-significant reduction in DBP [35]. Although the decrease in the DBP of the CMT group was statistically non-significant in this study, if given a longer intervention period, there would be a more obvious result, as most studies have confirmed a significant reduction in DBP following 8 or more weeks of continuous exercise in hypertensive and normotensive adults [32,36]. Interestingly, in the current study, within a time frame of 5 weeks, HIIT was shown to be effective in reducing DBP significantly. Therefore, HIIT could be a better option in controlling the DBP of pre-hypertensive young adults.

MAP measures the pressure necessary for the adequate perfusion of the organs of the whole body. Therefore, it could be a better indicator of perfusion than SBP. High MAP can be detrimental, leading to morbid conditions such as ventricular hypertrophy, myocardial infarction and stroke. HIIT intervention in this current study also demonstrated significantly greater reductions in the MAP in comparison to CMT (3.05 vs. 1.49). Similar findings were reported in past studies, wherein HIIT led to notable reductions in the MAP among pre-hypertensive subjects [37] and sedentary individuals [38,39]. Overall, the HIIT exercise resulted in a significant BP reduction and a favorable alteration in MAP, thus showing a positive cardiovascular response post-intervention. However, further studies are required to evaluate the potential mechanisms contributing to these physiological responses and changes in the pre-hypertensive population.

HIIT interventions are considered to be more effective and time-efficient interventions for BP and aerobic capacity level improvements as compared to other exercises [40]. Wahl P et al. [41] found that HIIT stimulated a transient increase in the circulating levels of vascular endothelial growth factor and hepatocyte growth factor. Thus, it can be postulated that HIIT intervention reduces BP by actively promoting and stimulating the angiogenic factors. A study comparing HIIT and CMT [16] showed that HIIT is far superior in lowering BP compared to CMT due to three factors: improving cardiorespiratory fitness, hormonal response and nitric oxide response, which is a mediator of vasodilation in blood vessels that plays a major role in BP control. It has been stated that HIIT interventions that last for 4–12 weeks are able to produce a larger decrease in SPB (−3.63 mmHg) than other forms of exercise [40]. Previous studies [16,17,18] also support the finding that HIIT is superior to CMT in improving cardiorespiratory fitness and reducing BP among normotensive and hypertensive individuals, but its efficacy in reducing BP among the pre-hypertensive population requires further investigation. In the current study, there was a significant difference in the mean SBP across the three groups, HIIT, CMT and CON, as revealed by the ANOVA test. Further analysis via a post hoc test demonstrated a significant mean difference in the SBP between the HIIT group and CON group (*p*-value = 0. 010 < 0.05). However, there was an insignificant mean difference in the SBP between the CMT and CON group participants (*p*-value = 0.282 > 0.05). Additionally, HIIT was found to be effective in reducing the DBP significantly (*p*-value = 0.002 < 0.05). Although PA has been associated with reduced BP, there can be some variations due to different training modalities, exercise prescriptions, intensities, frequencies and durations of intervention [34]. Nevertheless, the current study clearly demonstrates that HIIT is superior to CMT in controlling the progression of pre-hypertension towards hypertension in Malaysian young adults.

Studies by Stephen PJ et al. [42] and Paula TP et al. [43] revealed that the DASH diet and sodium restriction have significant effect on the reduction in SBP, DBP and HR among hypertensive patients. A recent meta-analysis also revealed similar observations [44]. Therefore, a possible reason that there was no reduction in SPB or DBP in the CON group could be non-adherence to the diet protocol, even after weekly reminders via phone calls to the participants to ensure that they were strictly adhering to the regimen. Although HIIT and CMT groups were not instructed to follow the DASH diet and sodium restriction as the researchers aimed to determine the effectiveness of HIIT and CMT solely, a significant SBP reduction and a non-significant DBP reduction were observed among the participants of the CMT group, whereas in the HIIT group, both SBP and DBP were significantly reduced after 5 weeks. In conclusion, the study results indicate a higher efficacy of HIIT over CMT even in the absence of the DASH diet to control the resting BP in the pre-hypertensive young adults.

## 5. Strengths, Limitations and Recommendations

Due to the time constraint and limited resources, the researchers were only able to recruit 32 participants for this research. However, this was a hypothesis-generating study and differed methodologically; therefore, even with the limited sample size, this research provided a deeper insight into the cardioprotective role of exercise training in BP. Second, the HIIT and CMT groups each consisted of only 4 and 6 females in the respective groups, whereas the CON group consisted of all males. This is due to the fact that during the screening process, many female participants were under hypotensive or normotensive categories, and after screening, eligible participants were divided randomly into three groups. The dietary intake of the participants in the CON group may have also played a significant role regarding controlling BP, since it was not possible to directly observe and monitor their adherence to the DASH diet and sodium restriction. Therefore, future studies with a larger sample size, a longer intervention duration, and the stringent control of the DASH diet plan are highly recommended. Furthermore, HIIT with the DASH diet plan could be a better approach towards controlling pre-hypertension in a short period of time.

## 6. Conclusions

HIIT can effectively reduce both the SBP and DBP of healthy, physically inactive pre-hypertensive young adults, but CMT reduced only the SBP in this study. Therefore, HIIT could be a promising alternative intervention in BP reduction and thus could be functional in preventing the progression of pre-hypertension towards hypertension among physically inactive young adults.

## Figures and Tables

**Table 1:** Test of normality at baseline for SBP, DBP and MAP among 3 groups.

	Group	Statistic	*p*-Value
Pre-SBP mean	HIIT Group	0.960	0.780
	CMT Group	0.981	0.972
	CON Group	0.853	0.063
Pre-DBP mean	HIIT Group	0.970	0.890
	CMT Group	0.912	0.294
	CON Group	0.874	0.112
Pre-MAP mean	HIIT Group	0.989	0.995
	CMT Group	0.921	0.365
	CON Group	0.923	0.387

Shapiro–Wilk Test; Level of Significance: *p < 0.05*.

**Table 2:** All groups’ SBP, DBP and MAP mean (X) with standard deviation (SD).

	HIIT Group X ± SD		CMT Group X ± SD		CON Group X ± SD	
	Pre-Test	Post-Test	Pre-Test	Post-Test	Pre-Test	Post-Test
SBP (mmHg)	122.76 ± 2.65	119 ± 3.91	125.23 ± 3.76	123.67 ± 3.98	127.93 ± 5.09	128.37 ± 5.32
DBP (mmHg)	78.57 ± 5.36	75.63 ± 4.86	77.23 ± 4.54	75.73 ± 4.26	74.00 ± 6.23	73.60 ± 5.78
MAP (mmHg)	93.14 ± 3.46	90.09 ± 2.57	93.20 ± 2.89	91.71 ± 3.08	91.98 ± 4.62	91.86 ± 4.18

**Table 3:** Paired sample t-tests for SBP, DBP and MAP among the 3 groups.

		Paired Differences				
Groups		Mean	Std. Deviation	*t*	df	*p*-Value
CON Group						
Pair 1	Pre-SBP mean–post-SBP mean	−0.43	3.68	−0.37	9	0.718
Pair 2	Pre-DBP mean–post-DBP mean	0.40	3.35	0.38	9	0.714
Pair 3	Pre-MAP mean–post-MAP mean	0.11	2.50	0.14	9	0.892
CMT Group						
Pair 1	Pre-SBP mean–post-SBP mean	1.57	1.54	3.22	9	0.011
Pair 2	Pre-DBP mean–post-DBP mean	1.50	3.10	1.53	9	0.161
Pair 3	Pre-MAP mean–post-MAP mean	1.49	2.12	2.22	9	0.054
HIIT Group						
Pair 1	Pre-SBP mean–post-SBP mean	3.76	2.83	4.20	9	0.002
Pair 2	Pre-DBP mean–post-DBP mean	2.93	2.23	4.16	9	0.002
Pair 3	Pre-MAP mean–post-MAP mean	3.05	1.64	5.90 *	9	<0.0005

Paired sample *t*-test was performed; * Indicates statistically significant at 5% level of significance.

**Table 4:** Comparison of SBP, DBP and MAP mean difference across the 3 groups.

ANOVA						
		Sum of Squares	df	Mean Square	F	*p*-Value
SBP	Between Groups	69.72	2	34.86	5.02 *	0.014
	Within Groups	187.53	27	6.95		
	Total	257.25	29			
DBP	Between Groups	32.25	2	16.12	1.87	0.173
	Within Groups	232.47	27	8.61		
	Total	264.71	29			
MAP	Between Groups	43.08	2	21.54	4.76 *	0.017
	Within Groups	122.13	27	4.52		
	Total	165.21	29			

One-way ANOVA test was performed; * Indicates statistically significant at 5% level of significance.

**Table 5:** Post hoc test (Tukey test).

Dependent Variable	(I) Group	(J) Group	Mean Difference (I-J)	*p*-Value
SBP	HIIT	CMT	−1.83	0.282
		CON	−3.73 *	0.010
	CMT	HIIT	1.83	0.282
		CON	−1.90	0.258
	CON	HIIT	3.73 *	0.010
		CMT	1.90	0.258
MAP	HIIT	CT	−1.57	0.244
		CON	−2.93 *	0.013
	CMT	HIIT	1.56	0.244
		CON	−1.37	0.337
	CON	HIIT	2.93 *	0.013
		CMT	1.37	0.337

Post hoc (Tukey) test was performed; * Indicates statistically significant at 5% level of significance.

## Data Availability

Not applicable.
